# In-silico prediction of dislodgeable foliar residues and regulatory implications for plant protection products

**DOI:** 10.1038/s41370-024-00675-w

**Published:** 2024-04-27

**Authors:** Yi Shi, Kanak Choudhury, Xiaoyi Sopko, Sarah Adham, Edward Chikwana

**Affiliations:** 1 Department of Biostatistics & Health Data Science, Indiana University Richard M. Fairbanks School of Public Health, 1050 Wishard Blvd, Indianapolis, IN 46202 USA; 2 Corteva Agriscience LLC, 9330 Zionsville Road, Indianapolis, IN 46268 USA; 3 Corteva Agriscience LLC, Abingdon, OX14 4RY United Kingdom

**Keywords:** Dislodgeable Foliar Residues (DFR) Prediction, Post-application Exposure, Dermal Exposure, Worker Exposure, In-silico Model, Random Forest

## Abstract

**Background:**

When experimentally determined dislodgeable foliar residue (DFR) values are not available, regulatory agencies use conservative default DFR values as a first-tier approach to assess post-application dermal exposures to plant protection products (PPPs). These default values are based on a limited set of field studies, are very conservative, and potentially overestimate exposures from DFRs.

**Objective:**

Use Random Forest to develop classification and regression-type ensemble models to predict DFR values after last application (DFR0) by considering experimentally-based variability due to differences in physical and chemical properties of PPPs, agronomic practices, crop type, and climatic conditions.

**Methods:**

Random Forest algorithm was used to develop in-silico ensemble DFR0 prediction models using more than 100 DFR studies from Corteva Agriscience^TM^. Several variables related to the active ingredient (a.i.) that was applied, crop, and climate conditions at the time of last application were considered as model parameters.

**Results:**

The proposed ensemble models demonstrated 98% prediction accuracy that if a DFR0 is predicted to be less than the European Food Safety Authority (EFSA) default DFR0 value of 3 µg/cm^2^/kg a.i./ha, it is highly indicative that the measured DFR value will be less than the default if the study is conducted. If a value is predicted to be larger than or equal to the EFSA default, the model has an 83% prediction accuracy.

**Impact statement:**

This manuscript is expected to have significant impact globally as it provides:

- A framework for incorporating in silico DFR data into worker exposure assessment,
- A roadmap for a tiered approach for conducting re-entry exposure assessment, and
- A proof of concept for using existing DFR data to provide a read-across framework that can easily be harmonized across all regulatory agencies to provide more robust assessments for PPP exposures.

## Background

Post-application exposure to workers – individuals who enter an area previously treated, or handle crops that have been treated, by a plant protection product (PPP) as part of their employment – is expected to occur mainly via the dermal route and predominantly from contact with dried residues as they perform supported tasks [1, 2]. Worker re-entry activities depend on the crop and growth stage, and include hand harvesting, crop inspection, pruning, cutting, sorting, bundling, and other supported activities [3]. Other sub-populations like residents and bystanders could also be exposed if they enter a treated area, but since this exposure is not from work-related tasks, it is considered incidental. Re-entry/post-application external dermal exposure can be generically expressed mathematically as:

$$Dermal\,Exposure=f\{AR,DFR,\,TC,\,T\}$$

The application rate (AR) provides the amount of active ingredient (a.i.) applied per unit area. Transfer coefficients (TC) and activity duration (T) depend on the specific task and are usually provided as defaults by regulatory agencies. Even though these parameters can also be refined, the focus here is on refining the DFR component only. The dislodgeable foliar residue (DFR) is a measurement of how much dried active ingredient residue (µg of a.i./cm^2^ of leaf surface) can potentially be dislodged from the leaf surface to the skin or clothing if an individual enters a field that has been treated with a PPP. The DFR value is usually normalized for application rate when used in exposure calculations, for example, µg of a.i./cm^2^ of leaf surface/kg of a.i. applied/hectare (µg/cm^2^/kg a.i./ha).

When experimentally determined DFR values are not available, regulatory agencies use conservative default DFR values as a first-tier approach to assess post-application dermal exposures to PPPs. In the EU, the European Food Safety Authority (EFSA) uses a default value of 3 µg/cm^2^/kg a.i./ha to estimate DFR on Day 0 (DFR0) after the application, derived from a database with 55 studies [1, 4]. Based on an analysis of 19 DFR studies, the United States Environmental Protection Agency (US EPA) and Health Canada’s Pest Management Regulatory Agency (PMRA) recommend a default DFR0 value of 2.5 µg/cm^2^/kg a.i./ha, estimated based on 25% of the application rate [2, 5]. Additionally, when re-entry exposure is calculated based on the default DFR value, the US EPA requires a calculated margin of exposure (MOE) of 2X higher than the level of concern (LOC) to waive the DFR study [6]. These default values are based on significantly fewer DFR studies than what the agencies and registrants currently have access to, are very conservative, and potentially overestimate exposures from dislodgeable foliar residues [7].

For tier 1 risk assessments of post-application dermal exposures which are above the acceptable levels, a DFR study for the active ingredient on a specific crop or crop group is one of the studies that can be conducted to provide a higher-tier refined exposure assessment. Since foliar residues are generally highest soon after application, studies are conducted to determine DFR on Day 0 after application (DFR0) once spray has dried. The DFR0 value is then used to refine post-application dermal exposure assessment by providing a worst-case exposure scenario [8].

How much foliar residue is on the leaf surface, and thus available for dislodging, depends not only on the active ingredient’s chemical structure, physico-chemical factors, but also on weather conditions. In turn, these factors impact chemical-specific dissipation and transformation parameters like wash-off, volatilization, hydrolysis, photolysis, and biodegradation [2]. In addition to chemical-specific characteristics, dislodgeability of foliar residues will also depend on crop-specific parameters which affect how likely the chemical will interact with, and dissipate from, the leaf surface such as crop 3-D architecture, crop growth stage, leaf structure and texture [9]).

With the implementation of the new EFSA [1] guidance, DFR studies are anticipated to get increasingly more complicated, with existing studies potentially having less of a chance of fulfilling the new criteria. European Union (EU) member states are not always aligned on accepting bridging of DFR data, however, it is not feasible to run a full-scale field study for each formulated product, crop type, and geographical location as these types of studies can take more than a year to plan, conduct, and report as they are season dependent and costly. As such, the EFSA default DFR value was used as the basis of the random forest classification model to provide a robust option for the prediction of likely foliar residues before conducting a field study as a higher tier refinement option during registration of PPPs in the EU.

The random forest algorithm used to develop the classification and regression-type ensemble models for predicting DFR0 values takes into consideration experimentally-based variability that accounts for physico-chemical properties of the PPP, as well as crop and location-specific parameters, and thus these models can be used as refinement options across all regulatory agencies. We propose that these in-silico models can be used as decision-making tools for when to conduct or waive DFR studies, and the predicted values can be used as a second-tier refinement for regulatory submissions. A similar approach for predicting dermal absorption values using in-silico modeling in a tiered-approach was recently proposed using a regression-type random forest model [10].

The structure of this paper is as follows: Section “Data and Methods” describes the DFR study database, and the methods chosen to predict DFR0 values. The typical issue of imbalance is highlighted and tackled by an over-sampling technique. Section “Results and Discussions” compares random forest ensemble classification and regression-type models built on the original imbalanced dataset to those where the dataset was balanced by an over-sampling technique. Several performance metrics, including a confusion matrix, are employed to choose the best performing models. Important variables which have significant influences on DFR0 are later identified. Regulatory implications on risk assessment of PPPs are discussed in Section “Implications for regulatory risk assessment”.

## Data and methods

The R software (version 4.2.2) was used for data cleaning and statistical analyses. The *rfImpute* and *randomForest* functions from *randomForest* package were used to impute the missing data and build the models, while the *smote* function from *performanceEstimation* package was used to generate synthetic samples.

### DFR database

**Fig. 1:**
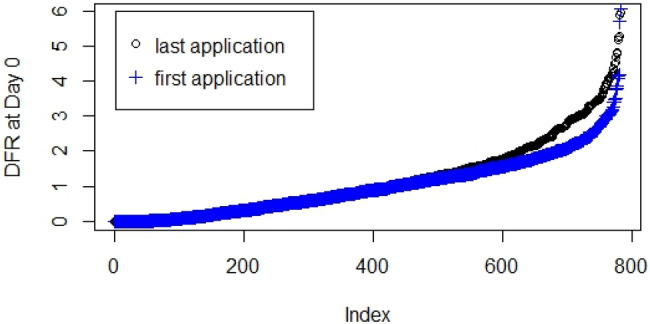
DFR values (µg/cm^2^/kg a.i./ha) at Day 0 after first application (crosses) and last application (circles) after residues have dried. The x-axis is an index of all the trials with multiple applications and had DFR values for both first and last applications.

The dataset used for this project consisted of data from 104 DFR studies on 28 active ingredients with registrations held by Corteva Agriscience^TM^. All studies were conducted according to US EPA’s Occupational and Residential Exposure Test Guideline OPPTS 875.2100 [11]. The products containing the a.i. were applied under representative conditions following Good Agricultural Practices (GAPs). Studies recorded DFR values at different times before and after application. Analyses of the dataset indicated that in cases where multiple applications were recommended, the magnitude of DFR values on day 0 after the first application were similar to DFR on day 0 (DFR0) after last application once spray had dried (Fig. 1). The correlation value R^2^ is 0.747, showing a relatively strong correlation between the first and last application. For DFR0 ≥ 3 µg/cm^2^/kg a.i./ha, DFR0 values after the last application were slightly greater than after the first application. Therefore, as a conservative approach, DFR0 values after the last application, without any correction for residues carried over from previous applications, were used for model building and analysis presented here.

Each of the 104 studies had 2–4 trial sites each, and about 3 replicates per site, for a total of 850 DFR0 data entries: 735 of them were from open field studies and 115 were from greenhouse studies. Ten of the trial sites did not have records for specific replicates but had records for average DFR value for that trial site, therefore the corresponding average value was imputed for each missing trial replicate value. Eight entries did not have records of either replicates or average values for DFR0 after last application, and there were no experimental notes explaining the missing values. Since all 8 missing entries had DFR0 values from the first application recorded, these were used in modeling since they were good estimates based on the similarity and strong correlations between the first and last applications as shown in Fig. 1. The 28 active ingredients were categorized into 11 formulation types, with the formulated products applied to 31 crops at 32 different trial locations across the United States (US), Canada, and Europe. Weather conditions for each observation day were available for all field studies, and the mean relative humidity and temperature on the day of last application was used in model building.

The univariate analyses demonstrated that the mean DFR0 on each crop varied from 0.114 to 4.968 µg/cm^2^/kg a.i./ha. The mean DFR0 at each location showed some variation across the different geographies where the studies were conducted, an indication of the influence of site-specific parameters on the magnitude of DFR0.

Based on an initial hypothesis that DFR0 is related to the active ingredient used, the crop/crop group, and location-specific variables, 14 parameters (Table 1) were considered in model building. These 14 parameters can be divided into three general categories related to the applied PPP, crop on which the PPP was applied, and site-specific properties.

**Table 1:** Summary of study parameters in DFR database.

Category	Parameters	N	Description
	DFR0	850	DFR values at Day 0 after last application; 780 DFR0 < 3 µg/cm^2^/kg a.i./ha, 70 DFR0 ≥ 3 µg/cm^2^/kg a.i./ha.
PPP	Active ingredient	28	Active ingredients monitored in the studies.
	Indication	3	Insecticide, Fungicide, Herbicide.
	Formulation Type	11	Aerosol, Emulsifiable Concentrate (EC), Emulsion in water (EW), Granular (GR), Microemulsion (ME), Suspension Concentrate (SC) Oil-based suspension concentrate (OD), Soluble Concentrate (SL), Water-soluble powder (SP), Water-dispensable granules (WDG), Wettable powder (WP)
	Formulation Category	3	Dry solid, Water-based, Organic solvent-based; Categories based on EFSA Dermal Absorption Guidance [22]
	Application Equipment	6	Aerosol, Air blast, Backpack, Granular Spreader, Ground boom, Handheld.
Crop	Crop type	31	Different crop/crop types the product was applied on.
	Crop Group	18	Classification based on similarities in agronomic practices. [1, 2]
	Leaf Texture	3	Hairy, Smooth, Waxy Based on Agricultural Re-entry Task Force (ARTF) classification [23]
	Crop Height	2	High, Low; classification was based on a threshold of 0.6 meter at time of application [1].
Site	Location	32	Geographical location where the study was conducted; 20 states in the US, 1 province in Canada, 8 countries in Europe.
	Location Index	13	Classifications in the US/Canada are based on EPA region index [2]; European countries are grouped into North EU and South EU residue zones based on EU SANTE/2019/12752;
	Field/Greenhouse	2	735 Field DFR0 values, 115 Greenhouse DFR0 values
	Relative Humidity	840	mean Relative humidity (%) at the last application day; 10 observations had missing humidity values.
	Temperature	850	mean Temperature (F) at the last application day

### Imbalanced classification

The EFSA Guidance assumes a default DFR0 value of 3 µg/cm^2^/kg a.i./ha in instances where experimentally determined DFR values are not available [1]. In the random forest classification model discussed below, the response variable (DFR0) was classified into two classes based on the EFSA default DFR0 value:

- DFR0 < 3 µg/cm^2^/kg a.i./ha is considered as negative (DFR0 Class = 0), and
- DFR0 ≥ 3 µg/cm^2^/kg a.i./ha is considered positive (DFR0 Class = 1).

**Fig. 2:**
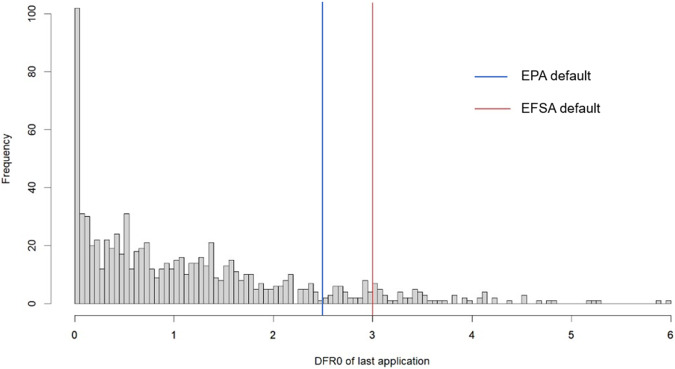
Histogram of DFR (µg/cm^2^/kg a.i./ha) at Day 0 of last application. The x-axis represents the DFR value (µg/cm^2^/kg a.i./ha), while the y-axis represents the frequency of that observed DFR0 in the database.

The histogram of DFR0 after last application shown in Fig. 2 indicates that the response is skewed with the imbalance ratio around 11:1, i.e., for every positive DFR0, there are 11 negative DFR0 values. It is expected to be skewed to the right since DFR follows a log normal distribution [12] with the EFSA default DFR0 value ranking in the 92^nd^ percentile of the dataset considered here, consistent with the EUROPOEM II data distribution [4] and reflecting the conservatism of the current regulatory approach. The US EPA value ranks in the 87th percentile of our dataset, also highlighting the conservatism in the US approach.

Most of the algorithms used for classification assume balance. Imbalance in the data lowers the prediction accuracy, especially for the minority class due to a lower sample size. Typically, the minority class is also of interest and requires a robust prediction accuracy. For the current dataset with the ratio of 11:1, the imbalance was remedied before modeling using the synthetic minority over-sampling technique (SMOTE).

SMOTE is one approach generally used to handle imbalanced classification problems by over-sampling the minority class “to create ‘synthetic’ examples based on the nearest *k* minority class neighbors instead of random sampling with replacement” [13]. The attractive feature of SMOTE is that the synthetic examples balance the original dataset, allowing the classifier to create larger and less specific decision regions which in turn can make the decision trees more generalizable and increase prediction accuracy of the minority class. For our analysis, SMOTE was used to generate a more balanced dataset for learning the predictor versus response relationship which was then compared with the model built on the original imbalanced dataset. There was a similar pattern of distribution in the original and SMOTE training set, with active ingredients which possessed low or high DFR0 values in the original set being reflected in the SMOTE set.

### Random forest imputation and model

Random forest is an ensemble learning method for both regression and classification that operates by constructing a multitude of decision trees at training time [14].

#### Imputation

In this study, random forest was used to impute any missingness based on similarity between each observation, rather than using any parametric model. There were 10 missing relative humidity values which were not recorded during the study and could not be determined from any official local weather logs for some of the greenhouse studies. After reviewing the study reports the missing values were predicted using other location variables since the missingness for these values was assumed to be missing at random (MAR) because the missing humidity values were related to other observations in the data [15].

#### Model

The complete dataset was randomly sampled into training and test sets stratified by the classes of DFR0. The training set contained 546 negative cases (DFR0 < 3 µg/cm^2^/kg a.i./ha) and 49 positive cases (DFR0 ≥ 3 µg/cm^2^/kg a.i./ha), accounting for 70% of the original dataset. The testing set had 234 negative cases and 21 positive cases, accounting for 30% of the original data. Both training set and testing set preserved the original imbalance ratio of classes (i.e., 11:1). A second training set was generated with a more balanced ratio of around 1.7:1 by using SMOTE on the original training set. A parameter of *k* = 5 was chosen to synthesize new examples based on the closest 5 neighbors. The new SMOTE training set had 588 negative cases and 343 positive cases.

**Fig. 3:**
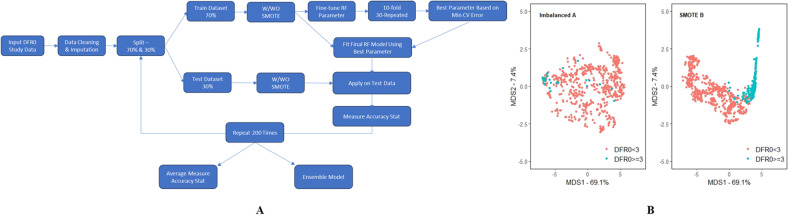
Random Forest Workflow for DFR prediction model development. Panel **A** is a schematic of the process followed for data processing and the machine learning workflow for development of methods A & B while Panel **B** is the multidimensional scaling (MDS) plots for the classification models based on the two methods. The axes for the MDS plot represent the first and second principal coordinates where these two principal coordinates explain 69.1% and 7.4% of the total variations of the factors.

Two methods were considered in developing the random forest classification and regression models; one based on the original imbalanced training set (Method A), and the other based on the SMOTE training set (Method B). The random forest model hyperparameters for each method were optimized by utilizing 10-fold and 30 repeated cross validation methods. All training samples were used to fit the random forest model and applied to the test set data based on the selected optimal hyperparameters. This whole process was repeated 200 times by randomly splitting the dataset into training (70%) and testing (30%) sets so that the training and testing samples represented different data structures, i.e., each of the 200 repetitions is essentially a different model as it contains a different set of training and testing datasets (Fig. 3A).

These 200 random forest models were used to build a final ensemble model based on majority voting for classification model, and average prediction for the regression model as detailed in Section “Results and Discussions”.

## Results and discussions

### Classification models

The algorithm discussed above, and outlined in the schematic represented by panel A in Fig. 3, was followed to build a random forest classifier on both imbalanced (Method A) and SMOTE (Method B) training sets, and the two methods were compared on the same testing set (Fig. 3B). Multidimensional scaling (MDS) is a common approach for graphically representing relationships between observations in multidimensional space [16]. Panel B in Fig. 3 shows the two-dimensional MDS plots for the two methods, demonstrating that observations clustered within their own groups better in Method B. In Method A, positive observations blended into the negative observations indicating it is not as good as Method B in distinguishing positives from negatives.

As shown in the schematic of the workflow in Fig. 3A, an ensemble model based on the majority class classification using 200 random forest models fitted on randomly selected training and testing sets was deployed to validate the predictive capabilities of methods A and B. Table 2 lists performance metrics for the two methods on the testing set as well as the ensemble model performance.

**Table 2:** Summary of performance metrics for the ensemble model classifiers based on test sets calculated from 200 partitions.

	Imbalanced (Method A)			SMOTE (Method B)	
		Observed Negative	Observed Positive	Observed Negative	Observed Positive
Confusion matrix	Predicted Negative	769 (90.47%)	27 (3.18%)	754 (88.71%)	12 (1.41%)
	Predicted Positive	11 (1.29%)	43 (5.06%)	26 (3.06%)	58 (6.82%)
AUC	0.911			0.930	
Balanced accuracy	0.800			0.898	
Specificity	0.986			0.968	
Sensitivity	0.614			0.829	

The ensemble model used test datasets from each of the 200 repeated partitions, with performance statistics showing significant improvement for the SMOTE-transformed dataset (Method B) compared to the original imbalanced dataset (Method A) except for specificity. Specificity and sensitivity are two frequently used metrics for model reliability. Specificity measures a classifier’s ability to identify a negative from true negatives (i.e., the model’s ability to identify as negative given that DFR0 is truly less than 3 µg/cm^2^/kg a.i./ha), while sensitivity refers to a classifier’s probability of correct “diagnosis” of positives (i.e., ability to identify a positive given that DFR0 is truly greater or equal to 3 µg/cm^2^/kg a.i./ha) [17].

The area under the curve (AUC) increased by 2% for SMOTE dataset compared to the original imbalanced dataset, indicating that Method B has a slightly favorable measure of separability. In the situation of an imbalanced dataset, balanced accuracy accounts for both positive and negative observations and reflects an average accuracy obtained from both the minority and majority classes. Specificity for Method B was slightly lower (2%) than for Method A, while sensitivity increased by 20% from Method A to Method B. In the case where both higher sensitivity and specificity cannot be achieved, higher sensitivity was prioritized, i.e., in exposure assessments it is less desirable to classify DFR values as less than 3 µg µg/cm^2^/kg a.i./ha when they are not (false negative), than getting a false-positive (classify as greater or equal to 3 µg/cm^2^/kg a.i./ha when measured value is actually lower than 3). After considering these performance metrics, the classifier built on SMOTE training set/Method B was preferred for building the regression model.

### Significant parameters

Gini impurity, a measurement of the diversity of a dataset, is one of the most popular algorithms for selecting the best split in decision trees and is often used to determine the importance of variables in model building [18]. In our case, Gini impurity is the probability of randomly choosing a pair of differently classified DFR0 values from the random forest classifier. The lower the Gini impurity is, the purer our dataset is after classification, and the better our classifier is. The measurement of variable importance used is the mean decrease of Gini impurity when a variable is chosen to split a node; the larger the decrease in Gini impurity, the better the model splits, thus the more important the variable is for predicting DFR0.

Method B (SMOTE) was used to identify the most important parameters from those listed in Table 1, based on their corresponding mean Gini decrease. The parameters that had the most impact on the predicted DFR0 values, in order of importance (mean Gini decrease provided in parentheses), were identified as: active ingredient (127), crop type (52), and location (51). Active ingredient was not only the most important variable, but also had a much larger mean decrease of Gini impurity compared with other variables, suggesting that the various physico-chemical properties associated with each active ingredient in the applied PPP influences DFR0 the most. It is also not surprising that crop-specific properties such as crop architecture, leaf type and texture, play an important role in how much of the active ingredient can be deposited and stick to the leaf surface. The geographical location’s impact on the magnitude of DFR0 values is tied to site-specific climate and weather conditions that influence mechanisms that affect stability, transformation, and dissipation of residues such as photolysis, volatilization, hydrolysis, and biodegradation. Other variables investigated had much lower and similar mean Gini decreases and the magnitude of their individual impacts could not be fully characterized based on our database.

### Regression model

Besides solving classification problems, random forest is also often used to solve regression problems to predict a numeric value, as shown by the successful efforts made to use random forest regression model to determine the leaf nitrogen content of oil palm [19] and chlorophyll levels in wheat [20] and tomato leaves [21]. Since DFR0 is a continuous variable, random forest can similarly be used to conduct a regression model to predict the magnitude of DFR0. The same algorithm used to build the classifier regression model was followed in building the random forest regression model using the SMOTE-generated dataset. The hyperparameters for the random forest regression model were optimized based on 10-fold 30 repeated cross validation steps. Mean squared error, mean absolute error and coefficient of determination, R^2^, were considered for the model performance. Additionally, the predicted value of the DFR0 was categorized into two classes based on the EFSA default DFR0 value (predicted DFR0 < 3 and predicted DFR0 ≥ 3) and then compared with the DFR0 class variable that was used for the classification model. A summary of performance metrics for the ensemble regression models based on the average prediction from the 200 random forest regression model are shown in Table 3.

**Table 3:** Performance metrics table for the ensemble regression model based on the average performance metrics from 200 repeated-sample random forest regression models.

	SMOTE (Method B)		
Mean squared error	0.2695		
Mean absolute error	0.3201		
R^2^	0.8244		
		Observed negative	Observed positive
Confusion matrix	Predicted negative	769 (90.47%)	17 (2.00%)
	Predicted positive	11 (1.29%)	53 (6.24%)
Specificity	0.986		
Sensitivity	0.757		
Negative predictive value (NPV)	0.978		
Positive predictive value (PPV)	0.828		

**Fig. 4:**
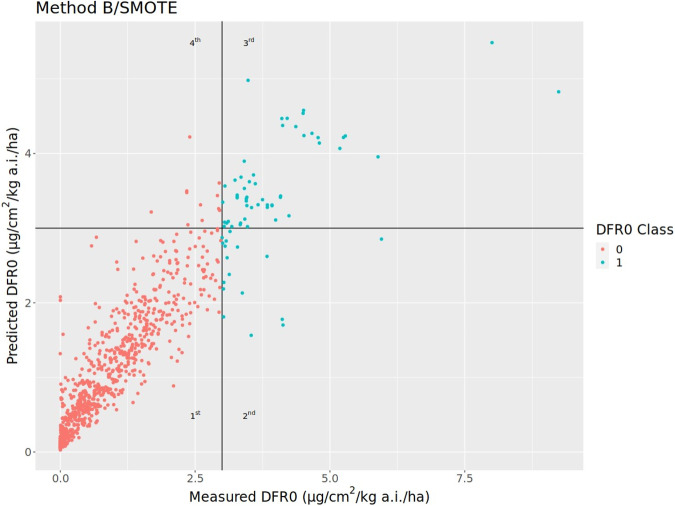
Plot based on the random forest regression model built on SMOTE training set and shows the correlation between predicted and measured DFR0 values (µg/cm^2^/kg a.i./ha). The lower left (1st) quadrant contains true negatives; lower right (2nd) quadrant contains false negatives. The upper right (3rd) quadrant contains true positives, while upper left (4th) quadrant contains false positives.

Table 3 shows that in this ensemble regression model the variation explained by the model (R^2^) is 82.44% for the SMOTE-transformed dataset (Method B), while Fig. 4 shows that the predicted DFR0 values from the preferred SMOTE-based ensemble regression model have a good alignment with the measured values. Most of the predictions cluster along the diagonal line in the 1st (true negatives) and 3rd (true positives) quadrants, indicating a high prediction accuracy of the regression model. From the 1st and 4th quadrants, if the regression model predicts that a DFR0 is less than the EFSA default value, there is about 98% predictive accuracy (NPV) that the measured DFR0 will be less than 3 µg/cm^2^/kg a.i./ha. Unfortunately, our database does not have as many studies with DFR0 greater than 3 µg/cm^2^/kg a.i./ha; which might be good from a product safety perspective, but it is not optimal for model building as it results in a lower prediction accuracy for DFR0 values higher than the EFSA default. From the 2nd and 3rd quadrants, if a value is predicted to be larger than or equal to the EFSA default, the model has about 83% prediction accuracy (PPV) that the measured DFR0 will be greater than 3 µg/cm^2^/kg a.i./ha. Based on our original dataset, the likelihood of getting a value above the EFSA default is low, and it is DFR0 values less than the default value which are more relevant to refining risk assessments and that is where the strength of the predictive tool lies.

## Implications for regulatory risk assessment

When these conservative default DFR values are used in conjunction with other higher percentile default parameters, the predicted re-entry exposures become truly over-conservative [7]. There are currently no available in silico models for DFR prediction, or a consistent regulatory framework, that provides an alternative approach to refine these default DFR values, besides conducting a full-scale DFR study. Using the vast amount of data that registrants and regulatory agencies have access to, the models presented here can be made more robust and further trained to provide the basis for more appropriate DFR0 values that could be used in regulatory submissions. New data can be generated specifically for scenarios with small sample sizes to increase the robustness of the model and minimize the imbalance in the dataset.

**Fig. 5:**
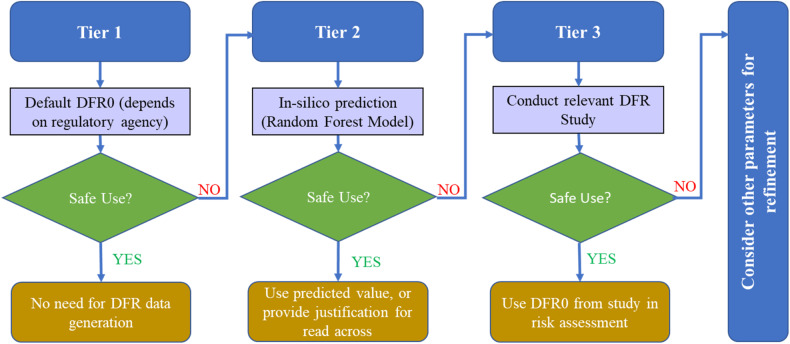
A proposed tiered use of DFR data in re-entry worker exposure assessment. Predicted DFR0 values can be used as Tier 2 refinement step before conducting a field study.

Statistical analysis from the proposed models indicated overall high alignment between predicted and measured DFR0 values suggesting that in-silico models such as the ones discussed here can be used in a tiered re-entry exposure assessment as shown in Fig. 5. What is considered Tier 1 is based on default parameters that each regulatory agency uses in the absence of experimentally determined DFR data. In the tiered approach proposed below, use of in-silico modeling can be used in a Tier 2 assessment to provide a basis for scientific justification of using read-across from previous studies, applying for DFR study waivers, and/or to predict DFR0 values that can be used in the risk assessment. If a safe use cannot be shown after all the relevant parameters are considered, then conducting the relevant DFR study should be considered to adequately characterize the re-entry exposure to that PPP.

Inclusion of a Tier 2 in-silico model approach allows for the use of predicted DFR0 values that better capture the larger range of PPPs, different agronomic practices, different crops/crop groupings, and variability in climatic conditions than what is currently provided by a single default value for all use scenarios and all PPPs. The proposed in-silico models not only provide estimates of DFR0, but also provide a data driven approach to refine regulatory defaults. As such, regulators and registrants can use these models to provide more realistic exposure estimates than those provided by current DFR0 default values.

## Supplementary Information

Reporting Checklist

## Data Availability

The datasets analyzed for model development are proprietary studies but are available publicly in instances where they have already been submitted to regulatory agencies for product registration. In such instances, data summaries and regulatory interpretation are publicly available as part of the registration reports published by regulatory agencies or can be made available from the corresponding author on reasonable request.
